# Relationships between digestive efficiency and metabolomic profiles of serum and intestinal contents in chickens

**DOI:** 10.1038/s41598-018-24978-9

**Published:** 2018-04-27

**Authors:** Stéphane Beauclercq, Lydie Nadal-Desbarats, Christelle Hennequet-Antier, Irène Gabriel, Sophie Tesseraud, Fanny Calenge, Elisabeth Le Bihan-Duval, Sandrine Mignon-Grasteau

**Affiliations:** 1 BOA, INRA, Université de Tours, 37380 Nouzilly, France; 2 Département d’analyse chimique biologique et médicale, PPF Analyse des systèmes biologiques, Université de Tours, 37032 Tours, France; 3 GABI, INRA, AgroParisTech, Université Paris-Saclay, 78352 Jouy-en-Josas, France

## Abstract

The increasing cost of conventional feedstuffs has bolstered interest in genetic selection for digestive efficiency (DE), a component of feed efficiency, assessed by apparent metabolisable energy corrected to zero nitrogen retention (AMEn). However, its measurement is time-consuming and constraining, and its relationship with metabolic efficiency poorly understood. To simplify selection for this trait, we searched for indirect metabolic biomarkers through an analysis of the serum metabolome using nuclear magnetic resonance (^1^H NMR). A partial least squares (PLS) model including six amino acids and two derivatives from butyrate predicted 59% of AMEn variability. Moreover, to increase our knowledge of the molecular mechanisms controlling DE, we investigated ^1^H NMR metabolomes of ileal, caecal, and serum contents by fitting canonical sparse PLS. This analysis revealed strong associations between metabolites and DE. Models based on the ileal, caecal, and serum metabolome respectively explained 77%, 78%, and 74% of the variability of AMEn and its constitutive components (utilisation of starch, lipids, and nitrogen). In our conditions, the metabolites presenting the strongest associations with AMEn were proline in the serum, fumarate in the ileum and glucose in caeca. This study shows that serum metabolomics offers new opportunities to predict chicken DE.

## Introduction

The intense selection performed during the last decades for chicken production-related traits has led to enormous genetic progress when considering feed conversion ratio (FCR) and growth rate, with broiler chickens being slaughtered 19% earlier and requiring 24% less feed to reach the same weight in 2010 than in 1970^1^. This is progress when considering solely the purpose of increasing animal protein production at a lower price, which has been the goal of genetic selection in livestock until now. This improvement of FCR is due to a low feed intake compared to growth, reduced physical activity, and high metabolic efficiency. Nevertheless, this advance relies on the use of optimal feedstuffs in poultry diets. Since these feedstuffs are becoming more and more expensive and increasingly are in competition with human food, digestive efficiency (DE), a component of FCR that has been neglected until now, must also be improved.

The digestive efficiencies of energy (measured by apparent metabolisable energy corrected to zero nitrogen balance or AMEn), proteins, lipids, and starch have a genetic origin, with heritability estimated between 0.33 and 0.47 in chickens, and have been successfully selected for^2^. However, the only available way to assess DE is through digestive balance trials, which are time-consuming and constraining, because the animals are kept in individual cages. All this consequently limits its integration in genetic selection schemes. Using indirect indicators such as biomarkers would thus be useful, but the lack of knowledge about the molecular pathways controlling DE in monogastric animals is very obvious. To our knowledge, no extensive metabolomic profiling for DE has been performed despite the suspected strong connections with feed digestion.

The aim of our study was first to identify predictive biomarkers of AMEn using the serum metabolome, which can be obtained from live animals. The second goal was to unravel the metabolic pathways influenced by DE by analysing the co-variation of DE traits and the metabolome from ileal and caecal contents, since these compartments are located at the end of the intestine, i.e. where most of the nutrients are absorbed or fermented.

The birds came from an advanced inter-cross line (AIL) obtained by 8 generations of crosses between two chicken lines divergently selected for either high (D+) or low (D−) DE. This divergent selection led to a difference in AMEn reaching 40%, with no impact on the body weights after 7 generations of selection^3^.

## Results

### Bird Digestive Efficiency Traits

**Figure 1:**
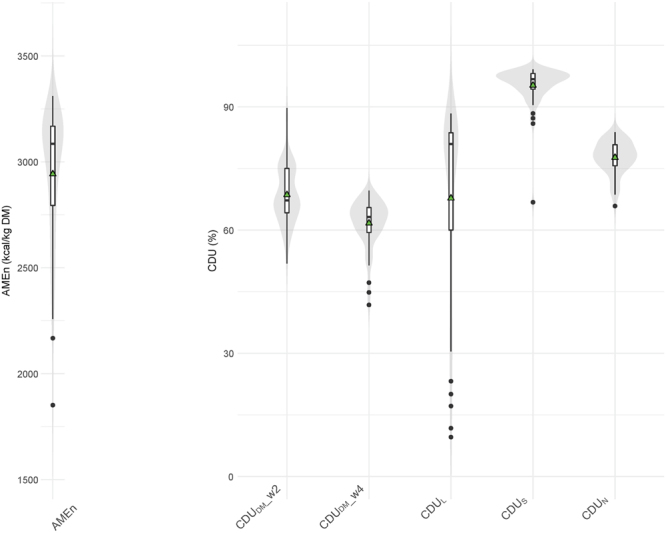
Distribution of phenotypic values of digestive efficiency traits for the 56 chickens selected for metabolomics (violin plots). AMEn: fecal apparent metabolisable energy corrected to zero nitrogen retention at 24–25 d, CDU_DM___w2_: fecal coefficient of digestive use of dry matter at 2 weeks, CDU_DM___w4_: fecal coefficient of digestive use of dry matter at 4 weeks, CDU_L_: fecal coefficient of digestive use of lipids at 4 weeks, CDU_S_: fecal coefficient of digestive use of starch at 4 weeks, CDU_N_: fecal coefficient of digestive use of nitrogen at 4 weeks. The means are indicated by a green triangle.

The chicken cohort studied was constituted from two subsets of 30 animals displaying extreme and opposite values of coefficients of digestive use of dry matter (CDU_DM_) measured at two weeks (63.4 ± 3.7% and 73.8 ± 5.1%; t-test p-value: 1.3 × 10^−12^). This bimodal distribution of CDU_DM_ at 14 days was not maintained for the coefficients of digestive use of DM, starch (CDU_S_), nitrogen (CDU_N_), lipids (CDU_L_), and AMEn at 4 weeks, as shown by the violin plots (Fig. 1) which include 56 birds (4 chickens with abnormal physiological or digestive traits or with missing DE feature values were not included). At 4 weeks, the faecal AMEn varied from 1851 to 3310 kcal.kg^−1^DM, while the average coefficient of digestive use ranged from 67.8 ± 23.3% for lipids to 95.3 ± 4.9% for starch. The weight at slaughter was about 707 ± 96 g and the feed conversion ratio between 15 and 28 days was 2.25 ± 0.46 g.g^−1^.

### ^1^H NMR Spectroscopic Profiles

**Figure 2:**
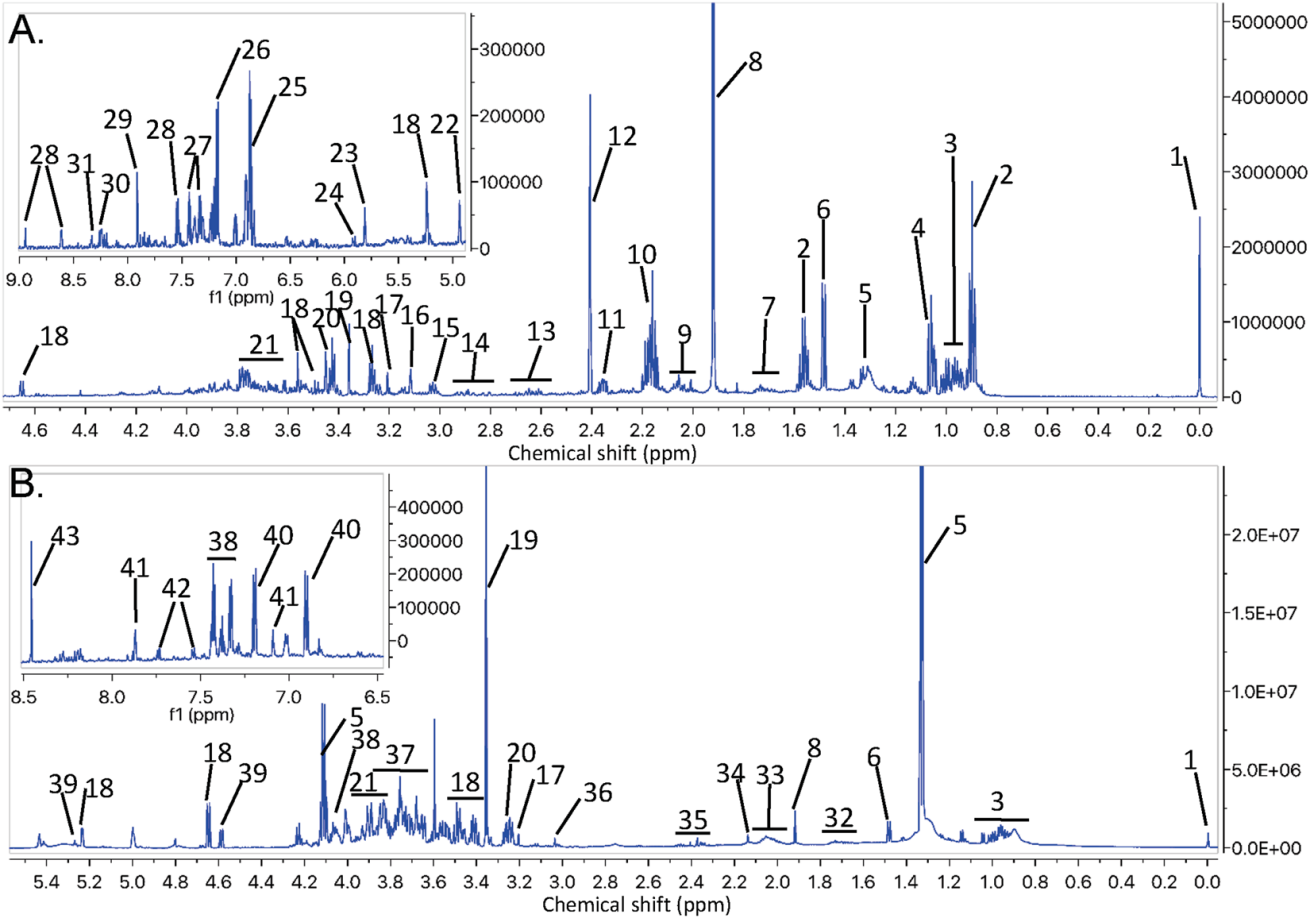
Representative spectra of (**A**) caecal and (**B**) ileal contents. 1. TSP; 2. Butyrate/valerate; 3. Ile/Leu/Val; 4. Propionate; 5. Lactate; 6. Ala; 7. Lys/putrescine/5-aminovalerate; 8. Acetate; 9. Pro; 10. Butyrate/propionate/valerate; 11. Glu; 12. Succinate; 13. 2-oxoisocaproate/Met/Asp; 14. Asp/3-(3-hydroxyphenyl)propionate; 15. Lys/5-aminovalerate; 16. Malonate; 17. Choline; 18. Glucose; 19. Methanol (extraction); 20. Glucose/Taurine; 21. Lys/Glu/Gln/Leu/Ala; 22. Xylose/galactose; 23. Uracil; 24. Uridine; 25. 3-(3-Hydroxyphenyl)propionate/4-hydroxyphenylacetate/4-hydroxybenzoate; 26. 4-Hydroxyphenylacetate; 27. Phenylacetate/Phe; 28. Nicotinate; 29. Deoxycytidine; 30. Nicotinate/deoxyadenosine; 31. Deoxyadenosine; 32 Leu/Lys/Arg; 33. Glu; 34. Met/Glu/Gln; 35. Glu/Gln/succinate; 36. Creatine; 37. Glucose/galactose; 38. Phe; 39. Galactose; 40. Tyr; 41. His; 42. Trp; 43. Formate.

The annotated representative ^1^H NMR spectra of the ileal content and caecal content extracts sampled at 26–27 d are shown in Fig. 2. The experimental and spectral data were deposited under the accession number MTBLS560 in the MetaboLights repository hosted by the EMBL-EBI (http://www.ebi.ac.uk/metabolights/MTBLS560).

#### Ileal content

The ^1^H NMR spectra of ileal content extracts at 26–27 d were divided into 160 buckets corresponding to 31 known metabolites and 71% of the buckets were assigned to one or more metabolites (the list of these metabolites is in Supplementary Figure S1). The intestinal content extracts contained mostly amino acids (Leu, Ile, Lys, Arg), lactate, and carbohydrates such as glucose and galactose.

#### Caecal content

The caecal content spectra at 26–27 d were divided into 191 spectral regions corresponding to 57 metabolites (Supplementary Figure S1; 74% of the buckets covered). Among them, some short-chain fatty acids (SCFA) were well represented (butyrate, valerate, propionate, formate, and acetate), as well as amino acids (mostly Ala, Val), carbohydrates (xylose, galactose, arabinose, glucose), and nucleosides (deoxycytidine, deoxyguanosine, deoxyadenosine).

#### Serum

The ^1^H NMR spectra of the sera polar fraction at 25 d were split into 64 adaptive spectral buckets corresponding to 35 different unambiguously assigned metabolites (Supplementary Figure S1; 94% of the buckets assigned). Organic acids (lactate), amino acids (Gln, Ala, Ser, Pro) and carbohydrates (glucose) were predominant.

### Partial Least Squares (PLS) Regressions: Predictive biomarkers of AMEn

According to their phenotype or their metabolomics principal component analysis (PCA) score, 6, 10, and 4 broilers were excluded from the metabolomic analyses for ileal content, caecal content, and serum, respectively.

The PLS regressions were adjusted to predict the AMEn value measured at 23–24 d from each metabolome.

#### Serum

**Figure 3:**
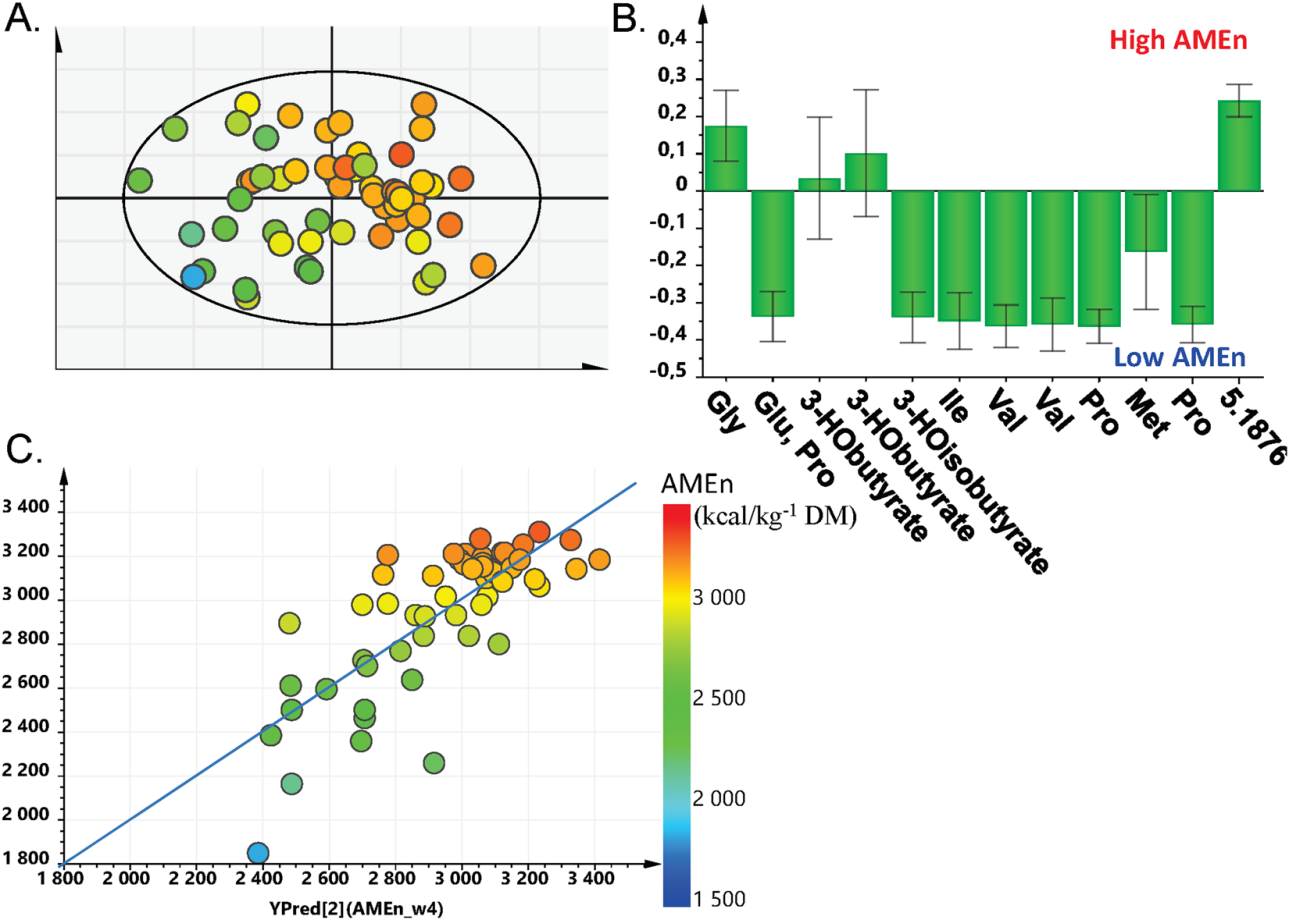
PLS regression from serum at 25 d (12 variables, 2 components, R^2^_X_ = 0.639, R^2^_Y_ = 0.591, Q^2^ = 0.48, CV-ANOVA = 1.12 × 10^−6^). (**A**) Score plot (t[z]: component z on the X space), (**B**) contribution of the variables through the loadings and their confidence interval (w*c[1]: X and Y loadings on component 1), and (**C**) predicted versus observed AMEn value (YPred: AMEn predicted, YVar: AMEn observed). 3-HObutyrate: 3-hydroxybutyrate, 3-HO-isobutyrate: 3-hydroxyisobutyrate, 5.1876: unknown metabolite with a chemical shift of 5.1876 ppm.

The fitted PLS regression from the serum, which requires the least invasive sampling technique, contained 12 spectral buckets selected according their regression coefficients on AMEn and importance in the projection (VIP). Those buckets corresponded to 8 known metabolites: glycine, glutamate, proline, 3-hydroxybutyrate, 3-hydroxyisobutyrate, isoleucine, valine, and methionine. The score plot, as well as the loadings with their confidence intervals derived from jackknifing, are presented in Fig. 3A,B, respectively. This regression model with two components explained 59% of the AMEn variability (R^2^_Y(cum)_) with a predictive ability of 0.48 (Q^2^_(cum)_) computed by 7-fold cross-validation and a CV-ANOVA of 1.12 × 10^−6^. The Root Mean Square Error of the Estimation (RMSEE), which indicates the precision of estimation of AMEn by the model, was 209.9 kcal.kg^−1^DM in the initial data set and 232.0 kcal.kg^−1^DM in the cross-validation data set. These results correspond to 7.1% and 7.9% of the average AMEn values, respectively (Fig. 3C).

#### Caecal content

A PLS regression with two components was retained to predict AMEn at 23–24 d. It was based on 17 buckets corresponding to 10 known metabolites and 6 unassigned spectral regions (Supplementary Figure S2). These metabolites were ketoisoleucine, 3-(3-hydroxyphenyl)propionate, glutamine, methylamine, ketoleucine, malonate, choline, galactose + lipids, 2-deoxyadenosine, and phenylacetate. The quality of this model was the best among the 3 fitted PLS regressions, with an R^2^_Y(cum)_ of 0.63, a Q^2^_(cum)_ of 0.51, and a CV-ANOVA of 9.03 × 10^−7^. The RMSEEs of the model were 202.4 kcal.kg^−1^DM (6.9% of mean AMEn) and 225.1 kcal.kg^−1^DM (7.6% of mean AMEn) on the initial and cross-validation data sets, respectively.

#### Ileal content

The ileal content at 26–27 d was less predictive of AMEn at 23–24 d than the serum and caecal content (R^2^_Y(cum)_ = 0.42, Q^2^_(cum)_ = 0.25, CV-ANOVA = 0.007). The PLS regression with two components included 8 buckets corresponding to amino acids (Leu, Lys, Arg, Pro, Val, Gln, Tyr), pyruvate, taurine, VLDL, and 2 unassigned buckets (data not shown).

### Canonical sPLS: Physiological markers of digestive efficiency traits

To give more insight into the biological pathways behind DE, a second approach without predictive purpose was used to assess, in a symmetric way, the relationships between two types of data sets: the DE traits at 23–24 d characterised by AMEn, CDU_S_, CDU_N_, CDU_L_ (Y) and a metabolomic data set (X). Canonical sPLS models with two components on X and Y were fitted. Using a LASSO (least absolute shrinkage and selection operator) penalisation, 40, 48, and 16 variables per component were selected for the ileal, caecal, and serum metabolome data sets, respectively. For each sPLS model that was fitted, only the first component was explicative of the DE traits. It explained 77%, 78%, and 74% of the variability of the DE traits for the ileum, caeca, and serum, respectively. Therefore, similarity matrices were computed based on the first component of the variables in both data sets. From these matrices, relevance networks, inferred from pairwise association scores between X and Y variables, were built using the network function of mixOmics to reveal correlation structures between the metabolites and the DE traits. These networks included 32 variables for the ileal content, 28 for the caecal content, and 15 for the serum after setting a cut-off at ± 0.5 on the association score. The association scores between these metabolomic variables and the DE traits are available in Supplementary Tables S1 to S3. Most of the variables in these networks were associated with the 4 DE traits. However, the coefficient of digestive use of starch was associated with 13% to 43% fewer metabolites than the 3 others in the 3 networks.

#### Global metabolic network

**Figure 4:**
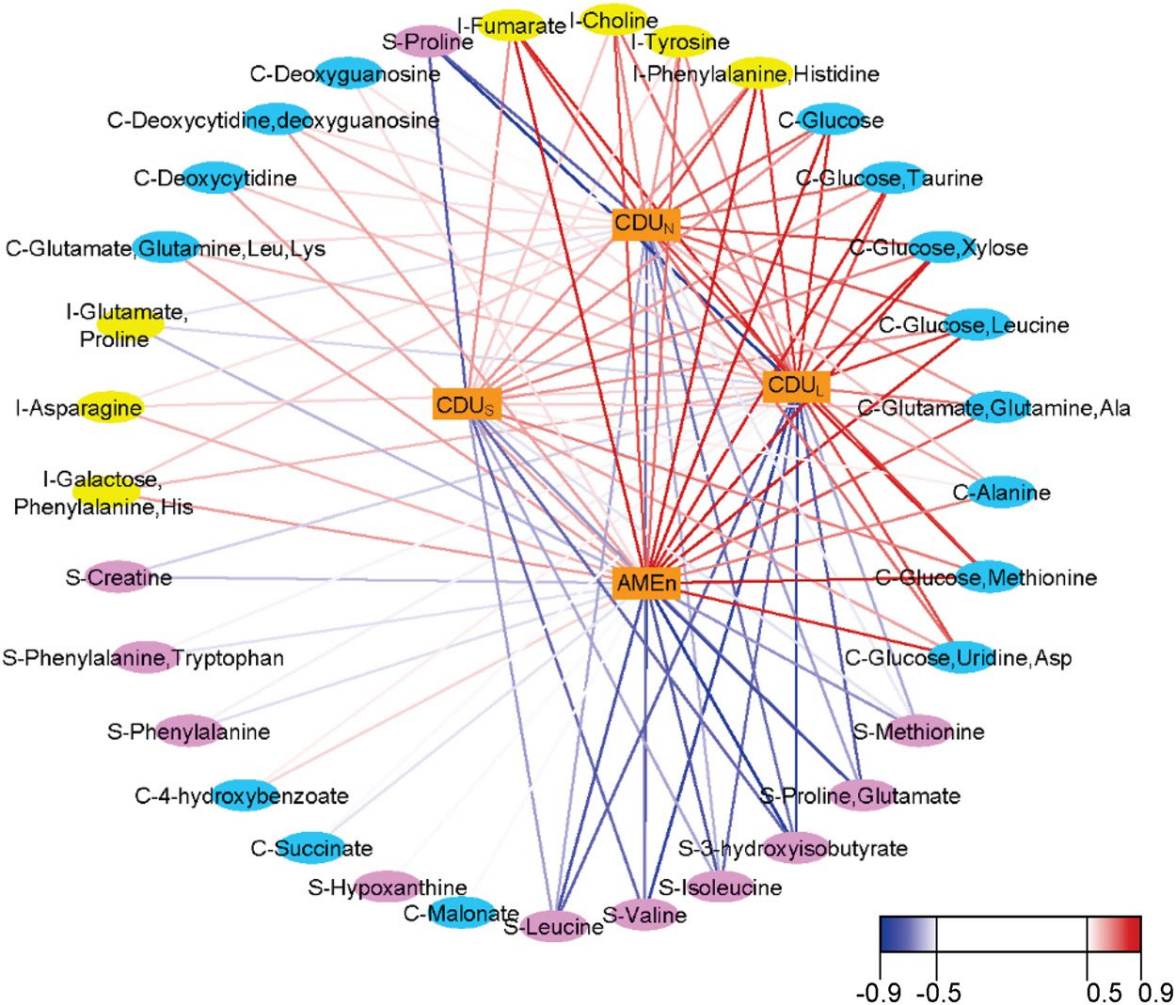
Global metabolic network between metabolites retained by sPLS and digestive efficiency traits (coefficients of digestive use of nitrogen, starch, lipids, and metabolisable energy at 4 weeks). This network combines metabolites from the ileal content (“I” or yellow node), caecal content (“C” or cyan node), and serum (“S” pink node). The edges are coloured according to the association score (cut-off at ±0.5).

The networks constructed for each metabolome were merged using Cytoscape into a single network without redundancy (i.e. all the buckets corresponding to glucose in caecal content were represented by only one bucket) and containing only the assigned metabolomic variables (Fig. 4). This global network included 11 nodes corresponding to metabolites from the serum, 7 from the ileum (mostly amino acids), and 15 nodes from the caeca (mostly carbohydrates and nucleosides). The association scores between the metabolomic variables and the DE traits represented by the colour of the edges peaked at –0.9 and 0.9. The AMEn had the most relationships with the metabolites. Its strongest associations were with proline in the serum (–0.94), fumarate in the ileum (0.85), and variables including glucose in caeca (0.86). The global network highlighted that the ileal and caecal contents buckets were mostly positively correlated with DE traits, while the serum ones were negatively correlated. Over the course of the construction of the global metabolic network, the number of buckets went from 48, 40, and 16 identified by canonical sPLS to 15, 7, and 11 buckets (nodes) in the global metabolic network corresponding to 16, 9, and 11 unique metabolites for the caeca, ileum, and serum, respectively.

#### Biological integration

A metabolite set enrichment analysis (MSEA) was performed using the 11 serum and 25 digestive metabolites (caeca and ileum) from the global metabolic network to identify the pathways enriched in metabolites associated with DE traits (Table 1). The serum and digestive metabolites were mostly representative of protein metabolism. However, the pathways of ammonia recycling and the urea cycle were also enriched in the digestive content. Most of the metabolites in those pathways were amino acids.

**Table 1:** Metabolite Set Enrichment Analysis performed on the list of 11 serum and 25 digestive content metabolites associated to DE derived from the global metabolic network.

Pathway	Match (a/b)	FDR p-value	Metabolites
**Serum**			
Protein biosynthesis	8/19	1.89 × 10^−10^	Phenylalanine, proline, isoleucine, leucine, methionine, valine, tryptophan, glutamate
Valine, leucine, isoleucine degradation	4/36	0.03	3-Hydroxyisobutyrate, isoleucine, leucine, valine
**Digestive content**			
Protein biosynthesis	12/19	5.24 × 10^−14^	Glutamate, tyrosine, phenylalanine, alanine, proline, asparagine, histidine, lysine, aspartate, glutamine, leucine, methionine
Ammonia recycling	5/18	0.0036	Glutamate, asparagine, histidine, aspartate, glutamine
Urea cycle	5/20	0.0042	Fumarate, glutamate, alanine; aspartate, glutamine
Aspartate metabolism	4/12	0.0047	Fumarate, asparagine, aspartate, malonate

In the match column, “a” corresponds to the number of metabolites observed by NMR in the pathway and “b” to the total number of metabolites implicated in the same pathway. Only the pathways with a p-value corrected by FDR lower than 0.05 were considered.

## Discussion

The study presented here took advantage of an advance intercross line (AIL) based on the D+ D− innovative animal model to evaluate the potential of metabolomics as a tool to easily assess and explain DE. Metabolomics is a powerful means to non-invasively detect subtle phenotypic changes or dietary responses, which are critical for farm trials involving animal selection and breeding, but this method has rarely been applied in livestock studies^4^. ^1^H NMR, which was used in our study, allows the measurement of the relative abundance of small molecules containing hydrogen atoms, such as the amino-acids, organic acids, carbohydrates, nucleosides, or short chain fatty acids.

The birds selected for this study were chosen for high and low CDU_DM_ measured at 2 weeks. It has been shown that CDU_DM_ at 3 weeks is genetically correlated with AMEn at the same age (r_g_ = 0.99)^3^. However, these two classes of CDU_DM_ were not conserved at 24–25 d, which indicated an adaptation (specific to each chicken) to the low digestibility diet during growth. In fact, some of the poor digesters selected at 2 weeks adapted to reach an AMEn ranging from 1741 to 3078 kcal.kg^−1^DM at 4 weeks, a range overlapping that of the birds not chosen for the metabolomic study and considered to be “medium” digesters. At the opposite end, the individuals with a high CDU_DM_ at 2 weeks still had high DE at 24–25 d, with an AMEn ranging from 3078 to 3310 kcal.kg^−1^DM.

A clear difference was observed between the metabolic profiles of the ileum and caeca, with a greater number of metabolites detected in the caeca (57 vs 31 metabolites in the ileum). This difference was previously observed by Gabriel *et al*.^5^ (with a slightly lower number of metabolites identified because of the use of 500 MHz NMR instead of 700 MHz) and could be related to a higher richness and diversity of microbiota in the caeca than in the ileum^6,7^. The metabolites identified in the ileum are mostly unabsorbed digestion products, while in the caeca they are mostly unabsorbed products of fermentation.

Among the metabolites identified, 23 were common to both digestive segments. They were mostly amino acids (13), but also carbohydrates (glucose, galactose), and taurine involved via bile acids in the intestinal absorption of lipids. Some other metabolites were recorded specifically in either the caecal or ileal contents. Indeed, 34 metabolites were found only in the caeca, among them nucleosides (deoxycytidine, deoxyguanosine, deoxyadenosine), pyrimidines (uracil, uridine), and carbohydrates (xylose, arabinose). Four amino acids (Arg, His, Trp, and Asn), as well as fumarate (Krebs cycle), creatine (synthesized in the liver from Met, Gly, and Arg), and dimethylamine were specific to the ileum. These are mostly hydrolysis and fermentation products of proteins, starch, and lipids, which is consistent as feed is thoroughly digested in the terminal parts of the small intestine.

The caecal and ileal contents are composed of digestion products as previously mentioned, but also of by-products from the direct or indirect synthesis or utilisation of compounds by the gut microbiota^8^. Choline, an essential nutrient present in the caeca and ileum is, for example, converted by anaerobic microorganisms into trimethylamine and dimethylamine, which are used as a carbon source by bacteria^9^. Taurine and organic acids/short-chain fatty acids (SCFAs) (lactate, acetate, formate, pyruvate) also shown to be present in the caecal and ileal contents may result, respectively, from the deconjugation of bile salts or mucosal cell secretion^10,11^ and nutrient conversion by the microbiota. Other carbon-containing molecules such as xylose and arabinose (released by complete hydrolysis of non-starch polysaccharides of vegetable origin) can be digested and subsequently fermented by the intestinal microbiota into other SCFAs (n-butyrate and propionate) that were specifically identified in the caeca^12,13^. Amino acid transamination or deamination involving reduction-oxidation (Stickland reaction) also produces butyrate and propionate after fermentation of the corresponding keto acids (keto-leucine, keto-isoleucine) by anaerobic bacteria in the caeca (e.g. proteolytic clostridia)^14^. SCFAs are common in chicken caecal contents with proportions dependent on the diet^15^. These SCFAs are used by the host. Chickens may obtain roughly 8% of their energy from SCFAs^16,17^. In addition, SCFA formation in the chicken caeca reduces the pH of the intestinal environment, which may inhibit acid-sensitive pathogenic bacteria. Among the SCFA, butyrate has gained particular interest, as it is the preferred energy source of the enterocytes and is known to regulate cellular differentiation and proliferation within the intestinal mucosa, thereby increasing intestinal tissue weight^16^. This is consistent with the fact that in D+ and D− lines, there is a link between DE and cell proliferation in the intestine^18^. Metabolites derived from vegetal compound degradation by the microbiota also seemed well represented in the caecal contents, including 3-(3-hydroxyphenyl)propanoate, 4-hydroxybenzoate, both products of phenolic degradation^8,19^, dihydroxyacetone, a product of plant fermentation, and putrescine that may result from pectin metabolisation by bacteria^20^. Amino acid derivatives produced by enteric bacteria were also present in the caeca as 4-hydroxyphenylacetate (Tyr), pyrrolidine (Pro), 5-aminovalerate (Lys), GABA (Glu), and nicotinate or vitamin B3 (Trp). The amino acids and their derivatives are partially related to the microbiota composition of the matrices^21^.

The serum metabolome is overall similar to the one described in Beauclercq *et al*.^22^ and not so different from the human metabolome, except for the glucose concentration, which is, as expected, in the order of 2 g/L in chickens.

The predictive model of AMEn based on the serum metabolomic profile included mainly amino acids (6). Five of them were associated with a low AMEn (Pro, Val, Ile, Met, Glu), while glycine was associated with a high AMEn. The elevated proportion of some circulating amino acids in the serum of low AMEn animals might be explained by difference in protein metabolism and amino acid utilisation compared with efficient birds. Indeed, it has been shown that D− broilers (low AMEn) have a heavier intestine, higher villi height, and more goblet cells, which suggests a higher protein deposition and turnover in the intestine^18,23^. The energy balance of these birds may also be affected given that metabolites from alternative sources of energy production such as 3-hydroxybutyrate and 3-hydroxyisobutyrate were in the model. The former is an indicator of ketogenic amino acid degradation^24^, and the latter a product of valine catabolism and a good gluconeogenic substrate^25^.

Furthermore, the predictive ability of the proposed model should now be tested in different conditions in order to explore whether this model is generic or dependent on chicken genotype and diet.

The canonical sPLS showed that each bucket was associated with the 4 DE traits (AMEn, CDU_L_, CDU_s_, and CDU_N_) most of the time, which is consistent with the high positive genetic correlations between those 4 traits^2^. We can conclude that similar molecular mechanisms between the digestive utilisation of lipids, carbohydrates, nitrogen, and metabolisable energy seem to be involved, or that the recorded metabolomes may not be able to discriminate between these mechanisms. Therefore, to make the discussion easier to read we will consider DE globally. To get a global picture of the pathways involved in DE, an enrichment analysis was performed on serum and digestive metabolites retained by canonical sPLS. However, this straightforward approach does not consider the role of the microbiota in the genesis of the metabolites. Currently, there exists neither a pipeline to investigate the biological relationship between serum and digestive content and nor methods considering the role of the microbiota in the digestive contents metabolome, which means that the biological information recovered from the metabolites related to DE was limited and partial.

The ileal contents had a set of 5 generic metabolites retained in the components of the canonical sPLS models that were highly and positively correlated with DE (>0.6). Among them 3 amino acids (Tyr, Phe, His) may come from the degradation of dietary proteins. The positive correlation between the ileal amino acids and the DE may be explained by a facility of efficient birds to hydrolyse proteins into a high amount of amino acids. The choline (vitamin B4), a supplement added to the feed but also a constituent of lecithin that is found in soy oil was correlated with DE in ileum contents, which may be an indicator of a better ability of the chicken selected for high efficiency to digest the feed or to a lower conservation due to difference in microbiota activity^9^. The last ileal metabolite positively correlated with DE was fumarate, a molecule involved in energetic metabolism. In the caeca, buckets containing glucose, amino acids or nucleosides were highly positively correlated with DE. The glucose may come from higher starch digestibility associated with the more developed gizzards in D+ broilers^3,26^ and with higher pepsin activity in the proventriculus^27^. The link between nucleosides (deoxycytidine, deoxyguanosine) and DE (positive correlation) may come from higher liberation of nucleoside in caeca due to higher renewal of intestinal mucosa, bacterial DNA or differences in the ability of caecal enterocytes to absorb nucleosides^28^. The serum metabolites retained in canonical sPLS (some amino acids and 3-hydroxyisobutyrate, a product of valine catabolism) were all negatively correlated with DE, the inverse of the observation for ileal and caecal contents, which suggested an abundance of amino acids in the terminal part of the intestine that have not been absorbed and therefore less likely to pass into the bloodstream.

However, these first attempts to understand and predict DE from blood and digestive metabolomes have some limitations. Indeed, the understanding of DE is limited by the lack of molecular knowledge about digestive metabolism in chickens. Combining NMR with mass spectrometry, which is becoming more and more common^29,30^, would make sense in this study to get a wider range of metabolic information and a more robust physiological integration. On the other hand, PLS regression highlighted several serum predictive markers of DE (AMEn) with good explicative and predictive abilities considering the complexity of this phenotype, which is influenced by genetics, diet, microbiota, and their interactions. These biomarkers are a first step in the metabolic characterisation of DE and need to be validated in independent populations.

In conclusion, the relationships between metabolomes and DE traits identified by canonical sPLS in this work could be useful to progress in the understanding of DE in chickens, as well as in monogastric animals. Furthermore, markers of AMEn identified by PLS regression may help in the development of more sustainable poultry production schemes by allowing a better combined nutritional and genetic approach to production in a context of a more variable and complex composition of broiler diets.

## Methods

All animal care and experimental procedures needed for this study were conducted in accordance with the French Animal Welfare Act and were approved by the Ethics Committee for Animal Experimentation of Val de Loire (Authorisation No. 01047.02). This ethics committee is registered by the National Committee under the number C2EA-19.

All chemicals were bought from Sigma Aldrich (Saint-Quentin Fallavier, France) unless otherwise specified.

### Birds and Sample Collection

This study used birds from the 8^th^ generation of an advanced intercross line (AIL) between two lines of medium growing chickens divergently selected either for low (D-) or high (D+) digestive efficiency (DE) between 21 and 24 d. The first generation of AIL (F1) was obtained by crossing D+ males with D− females and D+ females with D− males. Birds from following generations of AIL (F_n_ for generation n) were then obtained by crossing families of birds of generation F_n-1_. The selection criterion for DE was faecal AMEn on a difficult-to-digest, wheat-based diet at 3 weeks^2,31^.

Animals from the AIL were reared at UE PEAT (Pôle d’Expérimentation Animale de Tours, INRA, Nouzilly, France), on the floor during the first week and then in individual cages to measure DE. During the whole experiment, animals were fed ad libitum with the same diet as that used during the initial selection experiment, i.e. a diet adapted to nutritional requirements (2943 kcal.kg^−1^DM, 21% CP; 6% lipids), but of low digestibility due to its high content (55%) of Rialto wheat, a variety with high viscosity^32^. The diet composition is provided as supplementary Table S4.

**Figure 5:**
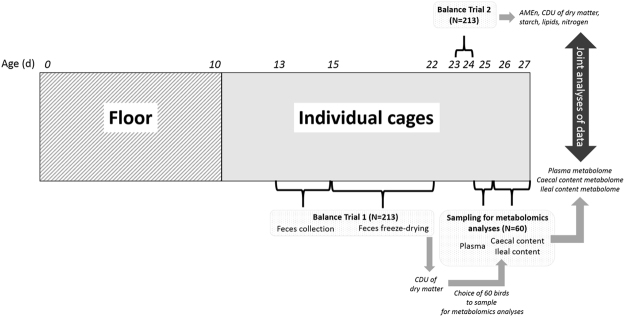
Experimental scheme.

Experimental protocol of measures is illustrated in Fig. 5. As the D+ and D− lines have been selected between 21 and 24 d, metabolome differences linked to DE are expected to be maximal around this age. As sampling caecal and ileal contents from the whole population (N = 213) was not possible, we had to select a subset of 60 birds with extreme DE values to sample for metabolome analyses. Taking into account the long time required for AMEn determination, it was necessary to find a proxy of AMEn at 3 weeks to select these birds. Previous analyses showed that coefficient of digestive use of dry matter (CDU_DM_) and AMEn were strongly correlated at 3 weeks^2^. We thus made a first balance trial between 13 and 15 d using a method based on total excreta collection, as described by Bourdillon *et al*.^33^. After faeces freeze-drying, we calculate CDU_DM_ at 2 weeks as a proxy of AMEn at 3 weeks as follows:

$$CD{U}_{DM}=\frac{F{I}_{DM}-F{W}_{DM}}{F{I}_{DM}}$$

where FI_DM_ is the dry matter feed intake between 13 and 15 weeks and FW_DM_ the dry matter faeces weight between 13 and 15 d.

Among the initial population (N = 213), we thus selected two subsets of 30 birds (males and females) displaying respectively the lowest and highest values of CDU_DM_ at 2 weeks.

After this preselection of animals, DE traits, blood, ileal and caecal contents were sampled between 23 and 27 d in order to build our models of relationship between DE and metabolomics at the same age, as close as possible to the age at selection. A second balance trial was thus done between 23 and 24 d to get faecal AMEn and coefficients of digestive use of dry matter (CDU_DM_), starch (CDU_S_), nitrogen (CDU_N_), and lipids (CDU_L_). In this second trial, gross energy, lipid, starch, and nitrogen content of individual freeze-dried excreta were measured for all birds using the Near Infrared Spectroscopy procedure (Foss, Hilleroed, Denmark) described by Bastianelli *et al*.^34^.

Blood was sampled at 25 d (end of the 2^nd^ trial) from the occipital sinus of the 60 selected fed birds and kept for 15 min at room temperature until coagulation. After centrifugation (3,000 g for 10 min), sera were aliquoted and stored at −20 °C until further analysis. At 26 and 27 days, the fed birds were killed by pentobarbital injection (0.5–1 mL/kg of live weight) in the occipital sinus. After slaughter, the content of distal ileum defined as the two third distal intestinal segment between the Meckel’s diverticulum and the ileo-caecal junction without the two last centimetres near the junction were sampled, homogenised and snap-frozen in liquid nitrogen. The content of both caecum were sampled as well and homogenised before being snap-frozen in liquid nitrogen. The ileal and caecal contents were stored at −80 C until extraction.

### Nuclear Magnetic Resonance Sample Preparation

Sera from the 60 selected birds were prepared for the high-resolution proton nuclear magnetic resonance (^1^H NMR) analyses by cold methanol precipitation of lipids and proteins. The unfrozen serum samples were centrifuged at 15,000 g for 10 min at 4 °C. After this, 450 μL of the supernatants were mixed with 1 mL of methanol and cooled at −20 °C for 20 min. The mixtures were then centrifuged as previously described, and 1,150 μL of the supernatants were collected in glass tubes for further solvent evaporation in a SpeedVac (ThermoScientific, Villebon sur Yvette, France) at room temperature and conserved at −20 °C^22^.

The extractions of polar metabolites from 100 mg ileal and caecal contents were performed on ice by the addition of 1 mL methanol with 4 mM of n-ethylmaleimide for thiol stabilization using gentle agitation to avoid breaking the bacterial cells^35^. The mixes were centrifuged (15,000 g, 2 min, 4 °C) before collection of the supernatants, which were stored at −80 °C until freeze-drying and further storage at −80 °C until use.

### NMR Spectroscopy Measurements

Before NMR analysis, the extracted serum samples were reconstituted in 220 µL pH 7.4 potassium phosphate buffer in 99% deuterium oxide (D_2_O) with 0.128 mM trimethylsilylpropionic acid (TSP) as an internal reference. The freeze-dried digestive content extracts were solubilised in 650 µL pH 7.4 potassium phosphate buffer in 99% D_2_O containing 10% v/v of TSP and 0.01% NaN_3_ (i.e. a bactericide). Both mixtures were then briefly vortexed and centrifuged at 4,000 g for 15 min at 4 °C to remove any insoluble components. The resulting serum supernatant or 600 µL of the digestive content was transferred to conventional 3 mm or 5 mm NMR tubes, respectively, for NMR analysis.

#### Serum ^1^H NMR

High-resolution ^1^H NMR spectra from the sera of the 60 birds were acquired on a DRX-600 Avance III HD spectrometer (Bruker SADIS, Wissembourg, France), operating at 600.13 MHz, with a TCI cryoprobe. NMR measurements were performed at 298 K. Standard ^1^H NMR spectra were acquired using a “cpmgpr-1d” pulse sequence with a relaxation delay of 25 s, an echo time of 80 ms (64 scans), and a time domain of 32,768 points. Water suppression was achieved by presaturation during the relaxation delay. Spectra were processed using Topspin version 3.2 software (Bruker Daltonik, Karlsruhe, Germany). The free induction decays (FIDs) were zero-filled to 65,536 data points, which provided sufficient data points for each resonance, and a line-broadening factor of 0.2 Hz was applied before Fourier transformation.

#### Ileal and Caecal Contents ^1^H NMR

The ^1^H NMR spectra of the ileal and caecal contents were recorded on a Bruker avance HD spectrometer equipped with a cryoprobe operating at 700.3 MHz. The acquisitions were performed at 298 K using a zgpr pulse sequence (64 scans, time domain 73,728 points, relaxation delay of 5 s). The spectra were processed using Topspin. The FIDs were zero-filled to 131,072 data points and a line-broadening factor of 0.3 Hz was applied prior to Fourier transformation.

#### NMR Spectra Post-processing

After manual correction of the phase distortion and baseline on all spectra, the ^1^H NMR spectra were imported into AMIX software (version 3.8.4; Bruker). The bin area method was used to segment the spectra between 0.6 and 9 ppm using the intelligent variable size bucketing tool. The spectral region containing water was excluded and a total of 64, 160, and 191 buckets were defined manually for serum, ileal content and caecal content spectra, respectively. Those bin areas corresponding to one or several metabolites were normalised by dividing each integrated segment by the total area of the spectrum on the 3 data sets.

#### Spectral Assignment

The ^1^H NMR spectra, referenced to the internal TSP chemical shift reference, were assigned using spectra from our in-house database and online databases, including LMDB (http://www.lmdb.ca) or the Chenomx NMR Suite 7.7 evaluation edition (Chenomx Inc, Edmonton, Canada).

### Chemometric Analyses

The distribution of the samples for DE traits (CDU_DM_, CDU_L_, CDU_S_, CDU_L_, AMEn) was assessed by violin plotting using the ggplot2 2.2.1 package with R 3.3.2.^36,37^. Violin plotting is a plotting method that combines a kernel density estimation showing the probability density of the data at different values and a boxplot displaying the quartiles, median, and outliers^38^. The DE traits, body weight, and feed conversion ratio values were expressed as mean ± standard deviation.

Principal Component Analyses (PCA) were performed on the 3 metabolomic data sets as exploratory unsupervised analysis. Individuals out of the 95% confidence interval of the Hotelling’s T-square were considered as outliers and excluded from the subsequent analyses^39^. The chemometrics approach proposed in this article was based on two partial least squares (PLS) algorithms. First, a PLS regression was used to predict the variable AMEn from each metabolomic data set independently. In a second step, for each metabolome, a sparse PLS canonical model was used to model bi-directional relationships between the data X (metabolomes) and the data Y (quantitative DE traits).

#### Partial Least Squares Regression Analysis (PLSR)

The PLSR^40,41^ were performed using the SIMCA 13 Software (version 13.0, Umetrics, Umeå, Sweden) on the 3 data sets (i.e., serum ^1^H NMR, ileal content ^1^H NMR, and caecal content ^1^H NMR) scaled to unit variance to predict the quantitative variable AMEn. For each model, the selection of metabolomic variables was carried by iteratively excluding the variables with low regression coefficients and wide confidence intervals derived from jackknifing combined with low variable importance in the projection (VIP) until maximal improvement of the quality of the models was reached. The model quality was evaluated after 7-fold cross validation by R^2^_Y_ (goodness of fit), Q^2^ (goodness of prediction), RMSEE (Root Mean Square Error of Estimation), RMSECV (Root Mean Square Error of Cross Validation) parameters, and CV-ANOVA (Cross Validation-ANalysis of VAriance). CV-ANOVA is a diagnostic tool for assessing the reliability of PLS models; the returned p-value is indicative of the statistical significance of the fitted model^42^.

#### Sparse PLS Canonical Analysis (canonical sPLS)

The canonical sPLS method was applied to model the bi-directional or symmetrical relationships between metabolites from each metabolome (serum, ileal content, caecal content) and DE traits (AMEn, CDU_L_, CDU_S_, CDU_N_) using the mixOmics R package, version 6.1.3^43,44^. All data were scaled to unit variance. A LASSO penalisation was used on the pair of loading vectors to select the metabolomic variables in the canonical sPLS model. For each model, the number of variables retained in the model was set to one-quarter of the metabolomic variables. As our objective was to study the relationships between DE and metabolomes, no variable selection was performed on the DE traits variables. Thus, 3 canonical sPLS models were fitted with 2 components for metabolomic and DE traits data sets. Pair-wise association matrices between couples of variables from metabolome and DE traits data sets were computed from the first component of each model. Networks were inferred from these pair-wise matrices using the network function in the mixOmics package^45^. Three graphs were built in which every node of metabolites variables selected was connected to nodes of the other DE traits variables by edges when the estimated correlation coefficients were greater than 0.5 in absolute value. The pair-wise association scores for each network were used as an input into Cytoscape 3.5.1^46^. Thereafter, these 3 networks were merged into a single one. A final reduced network was built by removing the unassigned spectral regions and by choosing a representative node for several buckets for the same metabolite inside the same biological compartment using the union parameter of the merge function of Cytoscape. The nodes (metabolites and DE traits) were plotted according to their number of edges and the intensity of the association scores.

### Biological integration

The serum and digestive metabolites retained as associated with DE in the global metabolic network were analysed by metabolite-set enrichment analysis (MSEA)^47^ implemented in Metaboanalyst (MetaboAnalyst 3.0, http://www.metaboanalyst.ca)^48^. The metabolite enrichment analysis of serum and digestive sets of metabolites was performed using over-representation analysis (ORA) with the “pathway-associated metabolite sets” provided, containing 88 metabolite sets based on normal metabolic pathways. The ORA was implemented using the hypergeometric test to evaluate whether a particular metabolite set was more represented than expected by chance within the given compound list. A cut-off was chosen at a FDR p-value of 0.05 to retain the most significantly enriched metabolic pathways.

### Data availability

The data sets generated during the current study are available in the Metabolights repository, http://www.ebi.ac.uk/metabolights/MTBLS560.

### Accession code

MTBLS560.

## Electronic supplementary material

Supplementary information
