# Supplementary material for: Relationships between digestive efficiency and metabolomic profiles of serum and intestinal contents in chickens
Source: Sci Rep. 2018 Apr 27;8:6678. doi: 10.1038/s41598-018-24978-9 (PMC5923279; doi:10.1038/s41598-018-24978-9)
Supplement: Supplementary file 1 — Supplementary information [file 41598_2018_24978_MOESM1_ESM.pdf]

Relationships between digestive efficiency and metabolomic profiles of serum and intestinal contents in chickens

Stéphane Beauclercq[1], Lydie Nadal-Desbarats[2], Christelle Hennequet-Antier[1], Irène Gabriel[1], Sophie Tesseraud[1], Fanny Calenge[3], Elisabeth Le Bihan- Duval[1] & Sandrine Mignon-Grasteau[*,1]

[1]BOA, INRA, Université de Tours, 37380 Nouzilly, France

[2]Département d'analyse chimique biologique et médicale, PPF Analyse des systèmes biologiques, Université de Tours, 37032 Tours, France

[3]GABI, INRA, AgroParisTech, Université Paris-Saclay, 78352 Jouy-en-Josas, France

[*] Corresponding author: sandrine.grasteau@inra.fr

Common to caeca, ileum and serum (n = 19)	Common to caeca and ileum (n = 4)	Common to caeca and serum (n = 1)	Common to ileum and serum (n = 6)	Present only in caeca (n = 33)	Present only in ileum (n = 2)	Present only in serum (n = 9)
Acetate	Aspartate	Glycine	Arginine	1,3-dihydroxyacetone	DMA	3-hydroxybutyrate
Alanine	Galactose		Asparagine	2-deoxyadenosine	VLDL	3-hydroxyisobutyrate
Choline	Taurine		Creatine	3-(3-hydroxyphenyl)propionate		Betaine
Formate	TMA		Fumarate	4-hydroxybenzoate		Citrate
Glucose			Histidine	4-hydroxyphenylacetate		Dimethylsulfone
Glutamate			Tryptophan	5-amino-valerate		Hypoxanthine
Glutamine				Acetamide		Myoinositol
Isoleucine				a-ketoglutarate		N,N-dimethylglycine
Lactate				Arabinose		Serine
Leucine				Butyrate		
Lysine				Creatinine		
Methionine				Deoxycytidine		
Phenylalanine				Deoxyguanosine		
Proline				GABA		
Pyruvate				Glycolate		
Succinate				Guanidoacetate		
Threonine				Ketoisoleucine		
Tyrosine				Ketoleucine		
Valine				Lipids		
				Malonate		
				Methylamine		
				n-butyrate		
				Nicotinate		
				n-valerate		
				Phenylacetate		
				Propionate		
				Putrecine		
				Pyrrolidine		
				Sucrose		
				TMAO		
				Uracile		
				Uridine		
				Xylose		

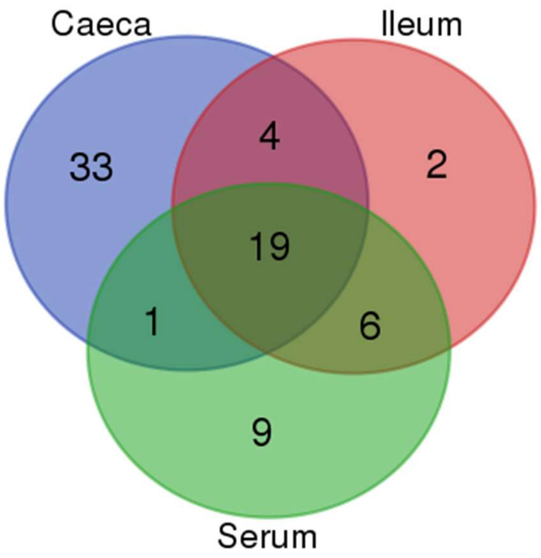

Supplementary Figure S1. Metabolites identified in ileum, caeca and serum contents spectra displayed in a Venn diagram

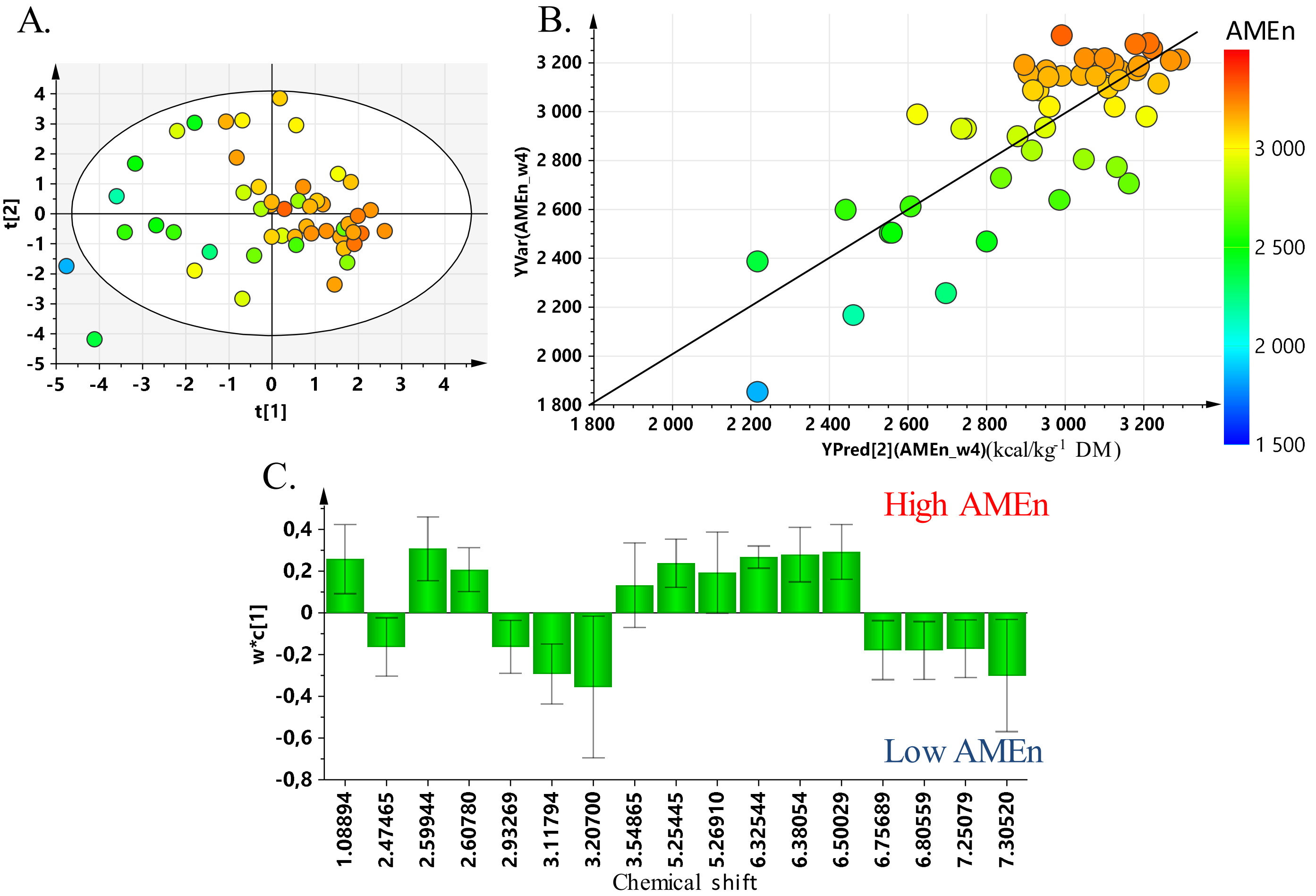

Chemical shift	Metabolite
1.08894	Ketoisoleucine
2.47465	3-(3-hydroxyphenyl)propionate, Gln
2.59944	Methylamine
2.60780	Ketoleucine
2.93269	
3.11796	Malonate
3.20700	Choline
3.54865	
5.25445	
5.26910	Galactose + Lipids
6,32544	
6,38054	
6.50029	2-deoxyadenosine
6.75689	3-(3-hydroxyphenyl)propionate
6.80559	3-(3-hydroxyphenyl)propionate
7.25079	
7.30520	Phenylacetate

Supplementary Figure S2. Regression PLS caeca model (17 variables, 2 components, $R^2_{Y(cum)} = 0.63$, $Q^2_{(cum)} = 0.51$ and CV-ANOVA = 9.03×10^{-7}). (A) Score plot, (B) predicted vs. observed plot, and (C) contribution of the variables through the loadings and their confidence interval. (t[z]: component z on the X space, w*c[1]: X and Y loadings on component 1, YPred: AMEn predicted, YVar: AMEn observed)

Chemical shift	Metabolite	AMEn	CDU_L	CDU_S	CDU_N
0.9332	Isoleucine	-0.78	-0.74	-0.63	-0.62
0.9646	Leucine	-0.79	-0.75	-0.65	-0.63
0.9903	Valine	-0.89	-0.85	-0.73	-0.72
1.0107	Isoleucine	-0.87	-0.82	-0.71	-0.69
1.0423	Valine	-0.90	-0.86	-0.74	-0.72
1.0678	3-hydroxyisobutyrate	-0.89	-0.85	-0.73	-0.72
1.9938	Proline	-0.94	-0.89	-0.77	-0.75
2.3493	Glutamate, Proline	-0.81	-0.77	-0.66	-0.65
2.6297	Methionine	-0.67	-0.63	-0.54	-0.53
3.0345	Creatine	-0.60	-0.57	0	0
3.3331	Proline	-0.90	-0.85	-0.74	-0.72
7.3282	Phenylalanine,Tryptophan	-0.55	-0.52	0	0
7.3754	Phenylalanine	-0.55	-0.52	0	0
7.4243	Phenylalanine	-0.57	-0.54	0	0
8.2035	Hypoxanthine	-0.51	0	0	0

Supplementary Table S1. Association scores from the network between serum ^{1}H NMR spectral regions (n = 15) and DE traits from canonical sPLS

**Chemical shift**	**Metabolite**	**AMEn**	**CDU_L**	**CDU_S**	**CDU_N**
1.1155		0.85	0.79	0.65	0.76
1.1328		0.93	0.87	0.72	0.84
1.1430		0.91	0.85	0.70	0.81
1.1526		0.88	0.82	0.68	0.79
1.1640		0.65	0.61	0.50	0.58
1.8377		0.72	0.67	0.56	0.65
2.3533	Glutamate, Proline	-0.61	-0.56	0	-0.54
3.0573	Tyrosine	0.70	0.65	0.54	0.63
3.0877	Tyrosine	0.84	0.78	0.65	0.76
3.1086		0.88	0.82	0.68	0.79
3.1178	Phenylalanine, Histidine	0.84	0.78	0.64	0.75
3.1278	Phenylalanine, Histidine	0.72	0.67	0.55	0.64
3.1557	Tyrosine	0.71	0.66	0.54	0.63
3.1716		0.72	0.67	0.56	0.65
3.2321		-0.72	-0.67	-0.56	-0.65
3.3276		0.79	0.74	0.61	0.71
3.5231	Choline	0.75	0.70	0.58	0.67
3.5404	Glucose	0.78	0.73	0.60	0.70
3.5540		0.73	0.68	0.56	0.66
3.9205	Asparagine	0.60	0.56	0	0.54
3.9769	Galactose, Phe, His	0.64	0.59	0	0.57
4.4402		0.74	0.69	0.57	0.66
4.5240		0.76	0.70	0.58	0.68
5.9216		0.87	0.81	0.67	0.78
5.9397		0.87	0.81	0.67	0.77
6.2560		0.84	0.78	0.64	0.75
6.3084		0.87	0.81	0.67	0.78
6.3254		0.87	0.81	0.67	0.78
6.5380	Fumarate	0.85	0.79	0.65	0.76
6.8364		0.74	0.69	0.57	0.67
6.8564		0.86	0.80	0.66	0.77
7.0182		0.60	0.56	0	0.54

Supplementary Table S2. Association scores from the network between ileum content ^{1}H NMR spectral regions (n = 32) and DE traits from canonical sPLS

Chemical shift	Metabolite	AMEn	CDU_L	CDU_S	CDU_N
2.4099	Succinate	-0.54	-0.51	0	0
3.1179	Malonate	-0.51	0	0	0
3.2498	Glucose	0.80	0.75	0.63	0.70
3.2624	Glucose, Taurine	0.80	0.75	0.63	0.70
3.4000	Glucose	0.85	0.80	0.67	0.75
3.4170	Glucose, Taurine	0.65	0.61	0.51	0.57
3.4711	Glucose, Xylose	0.85	0.80	0.67	0.75
3.4941	Glucose	0.83	0.78	0.65	0.72
3.5069	Glucose	0.83	0.78	0.65	0.72
3.7233	Glucose, Leucine	0.82	0.77	0.65	0.72
3.7605	Glutamate, Glutamine, Lys, Leu	0.63	0.59	0	0.55
3.7745	Glutamate, Glutamine, Ala	0.73	0.68	0.57	0.64
3.7804	Alanine	0.67	0.63	0.53	0.58
3.8405	Glucose, Methionine	0.86	0.80	0.68	0.75
3.8912	Glucose	0.84	0.79	0.66	0.73
3.9085	Glucose, Uridine, Asp	0.79	0.74	0.63	0.70
4.6510	Glucose	0.83	0.77	0.65	0.72
4.9306		0.61	0.57	0	0.53
5.2373	Glucose	0.82	0.77	0.65	0.72
5.8885		0.59	0.55	0	0.52
6.2844	Deoxycytidine	0.62	0.58	0	0.54
6.2975	Deoxycytidine, Deoxyguanosine	0.63	0.59	0	0.55
6.3151	Deoxyguanosine	0.70	0.66	0.55	0.62
6.5003	2-deoxyadenosine	0.55	0.51	0	0
7.6535		0.61	0.57	0	0.53
7.8097	4-hydroxybenzoate	0.56	0.52	0	0
7.8586	Deoxycytidine	0.58	0.54	0	0.51
7.9961	Deoxyguanosine	0.58	0.54	0	0.50

Supplementary Table S3. Association scores from the network between caeca content ^{1}H NMR spectral regions (n = 28) and DE traits from canonical sPLS

Raw material	Quantity (%)
Corn	6.04
Rialto wheat	52.5
Soy oil	6
Soy-meal	28.4
Corn gluten	3.1
Calcium carbonate ($CaCO_3$)	1.34
Dicalcium phosphate ($CaHPO_4$)	1.58
Salt (NaCl)	0.3
Premix oligo-vitamins[1]	0.35
DL-methionine	0.12
L-lysine	0.22
Coccidiostat (Diclazuril)	0.02

[1]Supplied per kilogram of diet: Co, 0.5 mg; Cu, 16 mg; Fe, 47 mg; I, 1.6 mg; Mn, 65 mg; Se, 0.2 mg; Zn, 72 mg; retinyl acetate, 12,000 IU; cholecalciferol, 3,440 IU; dl-α-tocopheryl acetate, 80 mg; thiamine, 4 mg; riboflavin, 6.4 mg; calcium pantothenate, 20 mg; vitamin B12, 0.02 mg; menadione, 4 mg; pyridoxine hydrochloride, 5.6 mg; folic acid, 0.4 mg; biotin, 0.24 mg; niacin, 80 mg; choline, 440 mg; and antioxidant, 40 mg.

Supplementary Table S4. Composition of the diet
